# The hypoglycaemia error grid: A UK-wide consensus on CGM accuracy assessment in hyperinsulinism

**DOI:** 10.3389/fendo.2022.1016072

**Published:** 2022-11-02

**Authors:** Chris Worth, Mark J. Dunne, Maria Salomon-Estebanez, Simon Harper, Paul W. Nutter, Antonia Dastamani, Senthil Senniappan, Indraneel Banerjee

**Affiliations:** ^1^ Department of Paediatric Endocrinology, Royal Manchester Children’s Hospital, Manchester, United Kingdom; ^2^ Department of Computer Science, University of Manchester, Manchester, United Kingdom; ^3^ Faculty of Biology, Medicine and Health, University of Manchester, Manchester, United Kingdom; ^4^ Department of Paediatric Endocrinology, Great Ormond Street Hospital for Children, London, United Kingdom; ^5^ Department of Paediatric Endocrinology, Alder Hey Children’s Hospital, Liverpool, United Kingdom

**Keywords:** hyperinsulinism, hypoglycaemia, accuracy, error grid, continuous glucose monitoring (CGM)

## Abstract

**Objective:**

Continuous Glucose Monitoring (CGM) is gaining in popularity for patients with paediatric hypoglycaemia disorders such as Congenital Hyperinsulinism (CHI), but no standard measures of accuracy or associated clinical risk are available. The small number of prior assessments of CGM accuracy in CHI have thus been incomplete. We aimed to develop a novel Hypoglycaemia Error Grid (HEG) for CGM assessment for those with CHI based on expert consensus opinion applied to a large paired (CGM/blood glucose) dataset.

**Design and methods:**

Paediatric endocrinology consultants regularly managing CHI in the two UK centres of excellence were asked to complete a questionnaire regarding glucose cutoffs and associated anticipated risks of CGM errors in a hypothetical model. Collated information was utilised to mathematically generate the HEG which was then approved by expert, consensus opinion. Ten patients with CHI underwent 12 weeks of monitoring with a Dexcom G6 CGM and self-monitored blood glucose (SMBG) with a Contour Next One glucometer to test application of the HEG and provide an assessment of accuracy for those with CHI.

**Results:**

CGM performance was suboptimal, based on 1441 paired values of CGM and SMBG showing Mean Absolute Relative Difference (MARD) of 19.3% and hypoglycaemia (glucose <3.5mmol/L (63mg/dL)) sensitivity of only 45%. The HEG provided clinical context to CGM errors with 15% classified as moderate risk by expert consensus when data was restricted to that of practical use. This provides a contrasting risk profile from existing diabetes error grids, reinforcing its utility in the clinical assessment of CGM accuracy in hypoglycaemia.

**Conclusions:**

The Hypoglycaemia Error Grid, based on UK expert consensus opinion has demonstrated inadequate accuracy of CGM to recommend as a standalone tool for routine clinical use. However, suboptimal accuracy of CGM relative to SMBG does not detract from alternative uses of CGM in this patient group, such as use as a digital phenotyping tool. The HEG is freely available on GitHub for use by other researchers to assess accuracy in their patient populations and validate these findings.

## Introduction

Congenital Hyperinsulinism (CHI) is a disease of recurrent, severe and unpredictable hypoglycaemia, with an estimated incidence of 1 in 28,000 births in the UK (1). Management of CHI relies upon detection and treatment of episodes of hypoglycaemia in children who may be completely asymptomatic. Since the first description of the condition in the 1950s (2), patients have relied upon self-monitoring of blood glucose (SMBG) by intermittent fingerprick testing to obtain knowledge about glucose levels. Unfortunately, this provides no trend information and there is a significant risk of missed episodes between tests, particularly overnight. Thus, over recent years, there has been a move towards the use of Continuous Glucose Monitoring (CGM) in CHI and other non-diabetic hypoglycaemia disorders to override problems inherent in SMBG (3).

Despite there being well established accuracy criteria for assessment and use of CGM in patients with diabetes (4), there are no such criteria for use in CHI. The CHI community has therefore relied upon routine measures such as mean absolute relative difference (MARD), hypoglycaemia sensitivity and occasional use of (diabetes specific) error grids to determine accuracy of CGM (3). There are three error grids that are used to report CGM accuracy. None were designed to asses CGM accuracy specifically and all are inappropriate for use in patients with CHI. The Clarke Error Grid (CEG) was developed in 1987 as a way of evaluating various blood glucose monitoring systems and analysing historical clinical data (5) but was criticised for its placement of risk boundaries and the small number of clinicians who informed its design (6). The Parkes Error Grid (PEG) was designed in 1994 and published in 2000 with the intention of assessing the clinical accuracy of various glucometers for patient use (7). It was considered an improvement over the CEG due to its focus on clinical risk rather than percentage accuracy and its development *via* a consensus of 100 clinicians. Finally, the Surveillance Error Grid (SEG) was published in 2014 (8) and based upon clinician responses to various clinical scenarios all involving patients with diabetes. This grid is mathematically complex and can only be readily interacted with on a designated website and in a limited way. The code for independent replication is not freely available, thereby limiting its use for comparative analysis.

**Figure 1:**
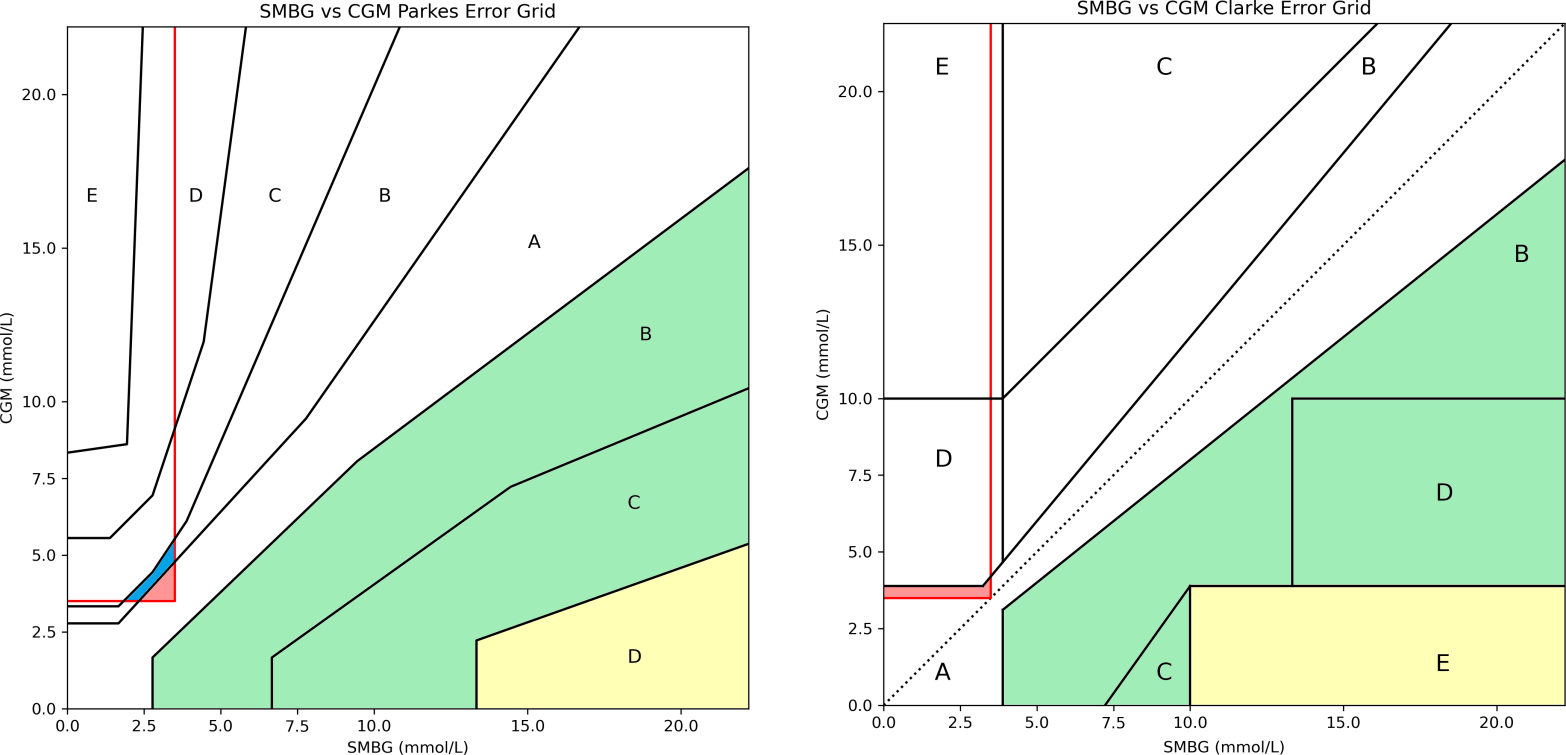
Parkes Error Grid (left) and Clarke Error Grid (right) demonstrating inappropriate risk areas for assessing CGM in CHI patients. For PEG: A = no effect on clinical outcomes, B = altered clinical action with little or no effect on clinical outcome, C = altered clinical action likely to result in altered outcome, D = altered clinical action could have significant medical risk, E =could have dangerous consequences. For CEG: A = values within 20% reference of the sensor, B = outside 20% but would not lead to inappropriate treatment, C = would lead to unnecessary treatment, D = potentially dangerous failure to detect hypo or hyperglycaemia, E = would confuse treatment of hypo for hyperglycaemia and vice versa. The red outlined box indicates false negatives for hypoglycaemia (<3.5 mmol/L).Both charts contain areas which categorise these false negatives as low risk (shaded blue) or no risk (shaded red) which, in reality, could be dangerous for a patient with CHI. Both charts also have large risk areas associated with missed hyperglycaemia which pose minimal risk (shaded yellow and green) to patients with CHI and could be re-categorised as risk A or B.

While the PEG and SEG may be improvements over the CEG, all three are designed to assess risk of glucose measurement errors in patients with diabetes and thus do not represent the risks faced by those with CHI. For example, all three report “very high risk” for a large under-reading at hyperglycaemia. This is entirely appropriate for patients receiving exogenous insulin therapy who might fail to administer the required dosage but poses minimal risk for a patient with CHI (**Figure 1**). Similarly, “no risk” is reported for measured values just above the hypoglycaemia threshold when the true value lies below this (false negative for hypoglycaemia). This situation would represent a potential risk for patients with CHI who are routinely advised by UK consensus that values above 3.5 mmol/L(63 mg/dL) are safe and below 3.5 mmol/L are not (9, 10) (**Figure 1**). Finally, all grids are designed to evaluate the difference between blood glucose meters and a gold standard rather than between CGM and SMBG. Thus, when CGM vs SMBG values are plotted on any of the established grids, the output does not accurately represent the risk posed to CHI patients using CGM for glucose measurement.

There are no studies reporting CGM accuracy in CHI using a standardised format. The first report on CGM accuracy in CHI used the FreeStyle Libre flash glucose monitor to show a MARD of 17.9% and a mean difference of +0.29 mmol/L (5.22mg/dL) based on 467 blood glucose readings with associated CGM values (11). Paired values were plotted on a SEG. The first report for the Dexcom system (G5) came in 2019 with 1155 paired values giving a MARD of 17.5%, a mean difference of -1.01 mmol/L (-8.09mg/dL) and no error grid analysis (12). Two more recent studies from mixed populations including some patients with CHI (60-64%) have shown MARD of 11.0% (13) and 13.1% (14) with one study plotting paired values on a CEG. These analyses all include multiple values of glucose >4mmol/L which are of great importance to a patient living with diabetes who may have to adjust insulin doses at higher glucose ranges but of limited practical interest to most patients with CHI who use CGM exclusively for hypoglycaemia detection. These analyses therefore likely overestimate the practical accuracy of devices at hypoglycaemia. Hypoglycaemia sensitivity is arguably the most useful measure of a device’s accuracy for patients with CHI as it focuses on the detection of hypoglycaemia (15). Reported results for hypoglycaemia sensitivity in CHI range from 43-73% (12, 13).

These studies do not describe how CGM and SMBG values were paired, thereby remaining ambiguous about pairing synchronicity and proximity of values. Further, the mean or maximum time difference between measurements and the mean absolute difference to understand error ranges are not consistently reported. Most importantly, these studies were not able to specify clinical risk of hypoglycaemia as they did not have access to an appropriate error grid.

Our aim was to therefore create a hypoglycaemia specific error grid with suitable CGM-SMBG pairing to assess the accuracy (and associated clinical risk) of a CGM device specifically for a patient with CHI or other hypoglycaemia disorder. We also aimed to use our newly developed error grid to evaluate the clinical risk of CGM inaccuracy in CHI using a Dexcom G6 and to set a baseline against which the accuracy of future devices can be tested.

## Methods

### Development of the hypoglycaemia error grid

In order to create our error grid, we followed the example laid out by Parkes et al (6) and administered a questionnaire to all UK based paediatric endocrinology consultants working regularly with patients with CHI in the two UK centres of excellence: Northern Congenital Hyperinsulinism Service (NORCHI) based at Royal Manchester Children’s Hospital (RMCH) and Alder Hey Children’s Hospital (AHCH); and Great Ormond Street Hospital (GOSH) Congenital Hyperinsulinism Service (16). Hypoglycaemia was predicated on a cut-off level of 3.5 mmol/L (63 mg/dL) as per UK consensus in the management of hypoglycaemia in patients with CHI (10, 17). The questionnaire asked respondents to define five blood glucose ranges (Appendix 1) and then assign levels of clinical risk (A: none, B: slight, C: moderate and D: severe) to hypothetical discrepancies between CGM and SMBG values. The full questionnaire is provided in Appendix 1.

**Figure 2:**
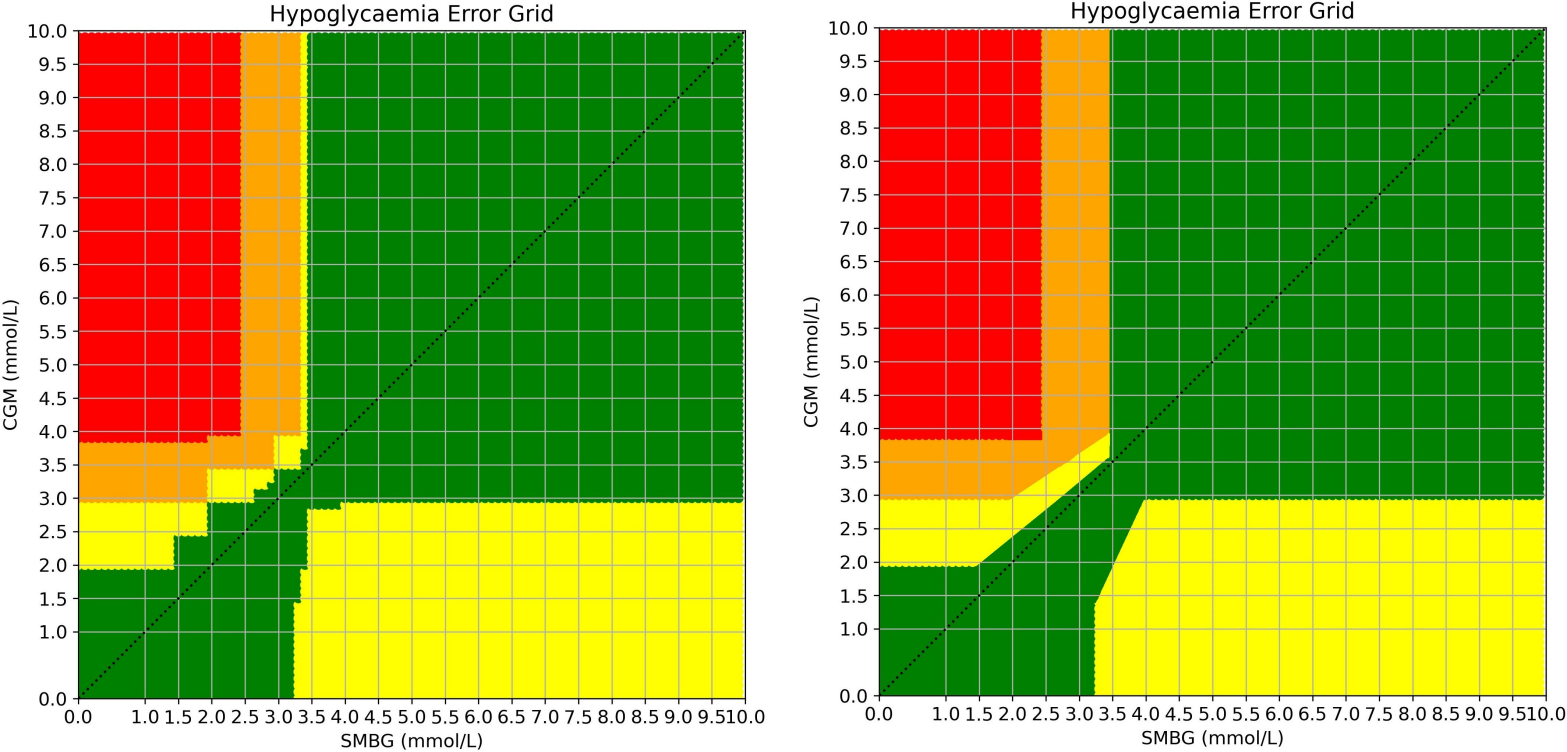
Demonstration of the averaged risk scores for all 10,000 combinations of SMBG vs CGM values before (left) and after smoothing (right). Risk levels: green = A, yellow = B, orange = C, red = D. The original grid generated by responses is demonstrated on the left with the smoothed grid on the right. For the final version, the slim vertical yellow column and the adjacent orange sections were combined. Staggered edges were converted to angled straight lines by consensus to aid usability and replication.

Following this, all questionnaires were mathematically collated. A grid was generated from 0.0 mmol/L to 10.0 mmol/L in 0.1 mmol/L increments on both the x and y axes for SMBG and CGM respectively, thus representing all possible 10,000 (100 x 100) combinations of CGM and SMBG values. Each respondent’s levels of risk (A, B, C or D) were plotted across the grid and a mean risk was generated for each of the 10,000 combinations. Most boundaries between risk levels were horizontal or vertical lines and were thus, not altered. Where staggered line boundaries occurred, the research team made decisions about where the straight boundary line should lie within the staggered line (**Figure 2**) in order to facilitate practical use of the grid. Straight lines of best fit were drawn through staggered edges and approved by consensus expert opinion to retain fidelity of clinical intrepretation.

### Accuracy assessment of Dexcom G6 CGM in patients with CHI

Patients with CHI were enrolled from the NORCHI service and all wore CGM (Dexcom G6) for 12 weeks. Of the 12 weeks for which patients wore CGM, the device was blinded for eight of these weeks. They were also asked to undertake at least two SMBG measurements per day (although not for calibration as the G6 device is factory calibrated) and whenever the CGM device reported a hypoglycaemia during the unblinded period. SMBG measurements were undertaken with a Contour Next One glucometer, independently verified as the home glucometer with the highest level of accuracy (18) and recommended by Dexcom for calibration of G5, a previous generation CGM device requiring calibration.

In order to maximize the interpretation of information, accuracy was assessed *via* two separate means. Firstly, a detection rate of hypoglycaemia was determined by examining all CGM values in a 30 minute time period (15 minutes before and after) around all SMBG detected hypoglycaemia. This enabled the interpretation of CGM as a predictive marker for hypoglycaemia over a diffuse time window instead of simple discrete point prediction. Separately, CGM and SMBG values were paired to assess for point accuracy to correlate with similar measures in other studies. Pairing of CGM and SMBG was restricted to a 5 minute window either side of the SMBG value to ensure close and suitable temporal matching of subcutaneous and blood glucose levels. This method ensured that CGM values always overlapped the SMBG measurement of interest. To obtain paired values, we wrote an analysis script in Python 3.8.8 which analysed each SMBG value and searched the CGM files for the closest value within five minutes before or after the SMBG and with a matching patient ID. Matched pairs were then assessed for various measures of accuracy and plotted on four separate error grids.

The study was undertaken under REC and HRA ethical approval (REC reference 07/H1010/88).

## Results

### Error grid creation

**Figure 3:**
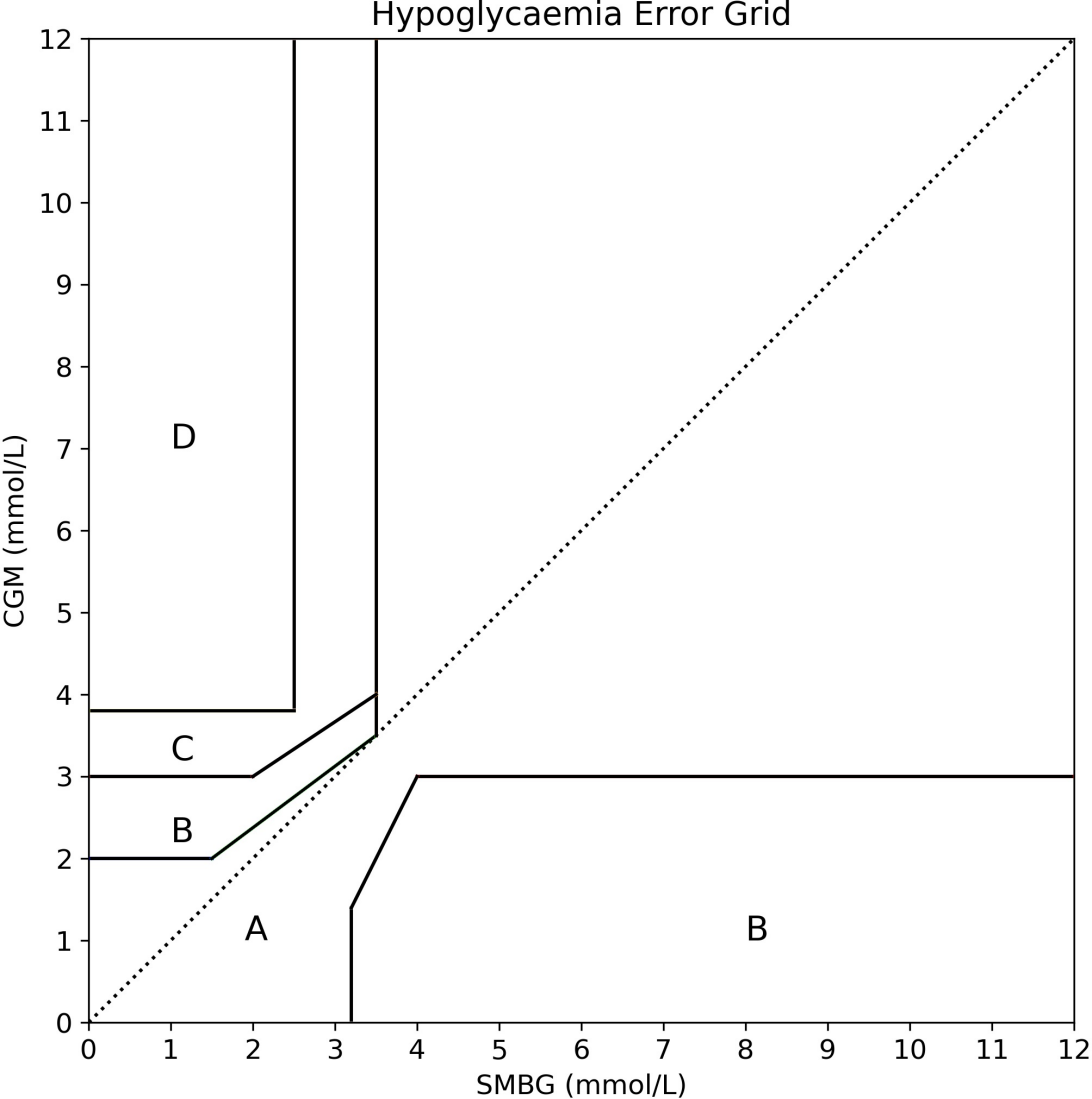
The new Hypoglycaemia Error Grid (HEG). Based upon the consensus opinion of 14 consultant paediatric endocrinologists working regularly with patients with CHI in the two UK centres of excellence. In contrast to error grids designed for use in diabetes, the HEG assigned relatively low risk to missed hyperglycaemia and relatively high risk to small errors around missed hypoglycaemia. As risks did not alter beyond 4.0mmol/L, the grid was extended from 10mmol/L to 12mmol/L with the same lines to incorporate the occasional value in this range.

Questionnaires were administered to, and returned from, all 14 paediatric endocrinology consultants working regularly with patients with CHI in the two UK centres of excellence. The Hypoglycaemia Error Grid (HEG) was generated as described and is freely available on GitHub (19) and displayed in **Figure 3**.

### Accuracy results

Ten patients were recruited to undertake regular SMBG readings and wear Dexcom G6 CGM device. Mean age was 8 years 11 months with a range from 21 months to 17 years and clinical details are provided in **Table 1**. Nine of the ten patients wore a Dexcom G6 CGM for 12 weeks and undertook regular fingerprick tests with a Contour Next One glucometer. One patient withdrew from the study after eight weeks due to complaints of painful sensor insertions and irritation with sensor alarms.

**Table 1:** Demographics and clinical details of all patients included in the study.

Patient	Age (years)	Gender	Time since diagnosis (years)	Genetic pathology	Medications
*Patient 1*	*14.5*	*Female*	*14.5*	*Paternally inherited KCNJ11 mutation + interruption of Chromosome 1*	*Diazoxide*
*Patient 2*	*3.2*	*Female*	*3.0*	*Not identified*	*Diazoxide*
*Patient 3*	*12.3*	*Male*	*11.9*	*Not identified*	*Diazoxide*
*Patient 4*	*5.4*	*Male*	*5.4*	*Maternally inherited dominant ABCC8 mutation*	*Diazoxide*
*Patient 5*	*3.1*	*Male*	*3.1*	*Homozygous ABCC8 mutation*	*Octreotide*
*Patient 6*	*3.4*	*Female*	*3.4*	*Maternally inherited dominant ABCC8 mutation*	*Diazoxide*
*Patient 7*	*17.3*	*Male*	*17.1*	*GLUD1 mutation (de novo)*	*Diazoxide*
*Patient 8*	*13.3*	*Female*	*13.0*	*Homozygous HADH mutation*	*Diazoxide*
*Patient 9*	*17.7*	*Male*	*7.4*	*GCK mutation (inheritance not determined)*	*Diazoxide*
*Patient 10*	*2.1*	*Male*	*2.1*	*Heterozygous HNF4A partial deletion*	*Diazoxide*

All patients continued on their mediactions for the duration of the study. Patient 6 withdrew from the study at the end of week 8 due to dissatisfaction with the CGM device.

Using a cutoff of 3.5mmol/L, CGM detected 96 of 188 SMBG detected hypoglycaemias, thus providing a detection rate of 51% over a 30 minute time window. When hypoglycaemia cutoffs were altered to 3.0mmol/L and 3.9mmol/L, detection rates were 50% and 68% respectively.

**Figure 4:**
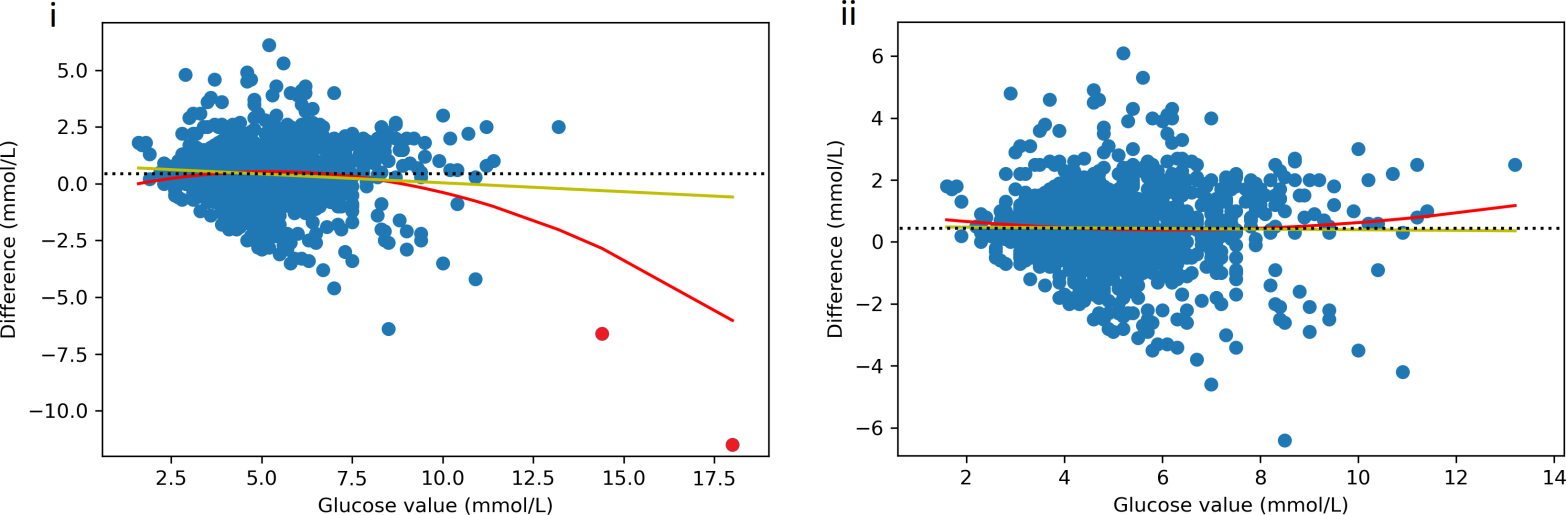
Scatter plot of difference (CGM minus SMBG) by glucose value. Yellow line = linear line of best fit, red line = 2^nd^ degree polynomial line of best fit, black dotted line = mean difference. Figure i) shows an inverse correlation between glucose value and difference with difference changing from positive to negative as glucose increases. However, when the two outliers (red) are removed, the difference does not vary by glucose value but remains positive across all glucose values (ii).

In total, 1441 paired readings were obtained from a possible 1,562 SMBG and 216,935 CGM readings with a mean absolute time difference of 1.3 minutes between CGM and SMBG readings. There were 185 SMBG values (13%) below 3.5 mmol/L in the paired dataset and 528 pairs where at least one value was <4mmol/L. The mean difference (CGM minus SMBG) between readings was +0.43 mmol/L, demonstrating that, on average, the Dexcom G6 reported a higher value than the SMBG. This difference reduced with increasing values of glucose (**Figure 4**(i)). However, when two outliers (>14mmol/L) were removed, the mean difference remained positive and static across all values of glucose (**Figure 4**(ii)). The mean *absolute* difference (MAD) was 0.93 mmol/L, demonstrating that the average error of a CGM reading was almost ± 1mmol/L.

**Figure 5:**
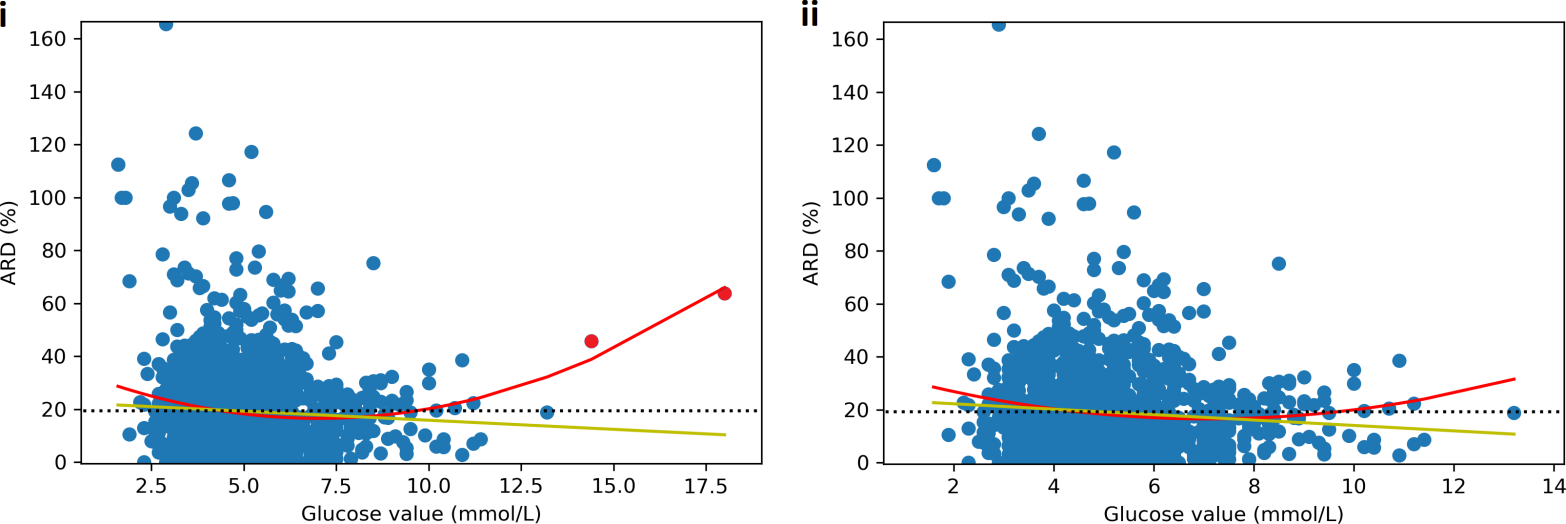
Scatter plot of absolute relative difference (ARD) between CGM and SMBG by glucose value. Yellow line = linear line of best fit, red line = 2^nd^ degree polynomial line of best fit, black dotted line = mean absolute relative difference (MARD). Figure i) shows a linear negative correlation between glucose value and ARD with ARD decreasing as glucose increases. However, the 2^nd^ degree polynomial shows a U shaped curve with the greatest accuracy in the normal glucose range from 5-9mmol/L. When the two hyperglycaemia outliers (red) are removed, the relationships are not significantly altered (ii).

Mean absolute relative difference (MARD) was 19.3% and absolute relative difference (ARD) varied by glucose value: highest accuracy (lowest ARD) was seen in the glucose range of 5-9mmol/L with an increase in ARD at values above and below this (**Figure 5**). Sensitivity and specificity were 43.8% and 92.4% respectively for hypoglycaemia (<3.5 mmol/L) and 39.6% and 95.7% for severe hypoglycaemia (<3.0mmol/L). When hypoglycaemia threshold was increased to 3.9 mmol/L, sensitivity and specificity were 52.6% and 89.1% respectively. While the hypoglycaemia sensitivity was low, the incidence of true hypoglycaemia when CGM was reading >4mmol/L and >5mmol/L was 4.2% and 0.96% respectively. **Table 2** presents the Odds Ratios for a true hypoglycaemia at various CGM device reported values.

**Table 2:** Odds Ratios (OR) for true (SMBG) hypoglycaemia at various CGM glucose values. n = number.

CGM reporting	n	OR true glucose <3.5mmol/L (n = 185)	OR true glucose <3.0mmol/L (n = 48)
>6.0mmol/L	401	0.007	0.002
>5.0mmol/L	729	0.010	0.001
>4.0mmol/L	1093	0.042	0.003
>3.5mmol/L	1250	0.079	0.010

As CGM values increase the odds of the true value being < 3.5 or 3.0 decreases. A CGM value >4mmol/L results in a 4% chance of a true value <3.5mmol/L and a 0.3% chance of a true value <3.0.

**Figure 6:**
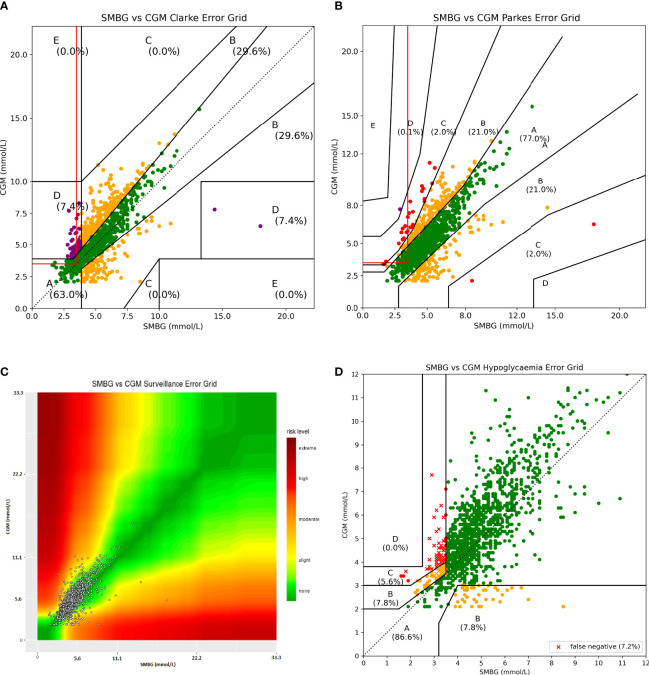
All four error grids plotted with 1441 paired values from CHI dataset. The red lined box in **(A, B)** indicates false negatives for hypoglycaemia. Several glucose datapoints in this box on the CEG (53) and PEG (84) would be classified as no risk but would potentially pose a risk to a patient with CHI. In plot **(C)**, due to a lack of colouring of the plots, categories are less defined than in a grid model. Data points appear clustered in the left lower corner as numerical values on x and y axis are immovable by software constraints. For the HEG **(D)** 4.7% of values have been classified as moderate risk to the patient; these represent an over reading by the CGM device at a time of hypoglycaemia (<3.5mmol/L). There are no false negatives classed as no risk. False negatives are differentiated in representation by being plotted as a cross rather than a dot.

Results plotted on the CEG, PEG, SEG and our HEG provided different results, as expected from the premise of our study. Using the CEG (**Figure 6A**), there were 53 datapoints (3.7%) that were classified as A (no risk) that were a false negative for hypoglycaemia. The percentage of points classed as low (B), moderate (C) and severe (D) risk was 29.6%, 0.0% and 7.4% respectively.

The PEG (**Figure 6B**) provided different results for the percentage of values within each risk category (**Table 3**). Using the PEG, there were 84 datapoints (5.8%) that were classified as no risk but which represented a false negative for hypoglycaemia. Data plotted on PEG were biased to report a lower risk with only 2.0% of values being classed as moderate or high risk (C+).

**Table 3:** Comparison of percentage risk allocated to each category between error grids.

	Percentage values falling into each risk category						
Error Grid	A (total)	A (false negative)	B	C	D	E	C+
**CEG**	63.2	3.7	29.6	0.0	7.4	0.0	7.4
**PEG**	77.0	5.8	21.0	2.0	0.1	0.0	2.0
**SEG**	65.0	??	23.6	11.2	0.2	0.0	11.4
**HEG (all)**	88.3	0.0	7.0	4.7	0.0	NA	4.7
**HEG (<4mmol/L)**	63.6	0.0	21.3	15.2	0.0	NA	15.2

HEG, Hypoglycaemia Error Grid. The HEG allocates a slightly lower percentage of values to categories of moderate and higher risk (C+) than the average over the charts due to the allocation of low risk to missed hyperglycaemia. However, this is reversed when the HEG is used only with pairs where at least one value is <4mmol/L and offers a practical risk of 15.2% of values categorised as C+. The CEG and PEG (and likely SEG) classify several false negatives for hypoglycaemia as no risk, while the HEG classifies all false negatives as at least low risk. It is not possible to quantify false negatives assessed by the SEG due to the limited way with which it can be interacted. ?? - not possible to compute, NA, not applicable.

Analysis of the results with the SEG was limited due to the restricted functionality of its use on a website. Requests were made for access to the underlying code so further analysis could be undertaken but unfortunately were not granted. We were thus not able to ascertain the number of false negatives for hypoglycaemia classed as low risk. However, some results were obtained, and these are shown in **Figure 6C** and **Table 3**. While the SEG did grade more results in category C, it labels this category as only “Slight, Higher risk” rather than the allocation given to C in the CEG and PEG which is “altered clinical action, likely to affect clinical outcome” and thus is hard to directly compare.

### Practical accuracy results

The above results are based upon all of the paired values in the available dataset, irrespective of the glucose value. While this offers a meaningful comparison with previous studies, and across various error grids, it does not provide the most practical assessment of accuracy for those living with CHI. All respondents to the HEG questionnaire provided 4mmol/L or lower as a cut-off above which patients did not need to actively manage their glucose. Four possible combinations of CGM and SMBG values are thus available:

1. False negative (SMBG <4, CGM >4)
2. False positive (SMBG >4, CGM <4)
3. True positive (SMBG <4, CGM <4)
4. True negative (SMBG >4, CGM >4)

Combinations 1 to 3 were all identified as being of interest to the patient with CHI and accuracy in these situations should be assessed. Because information about glucose is not required for insulin dosing for the majority of patients with CHI, combination 4 is rarely of interest and should not be included in an assessment for fear of overestimating practical accuracy.

**Figure 7:**
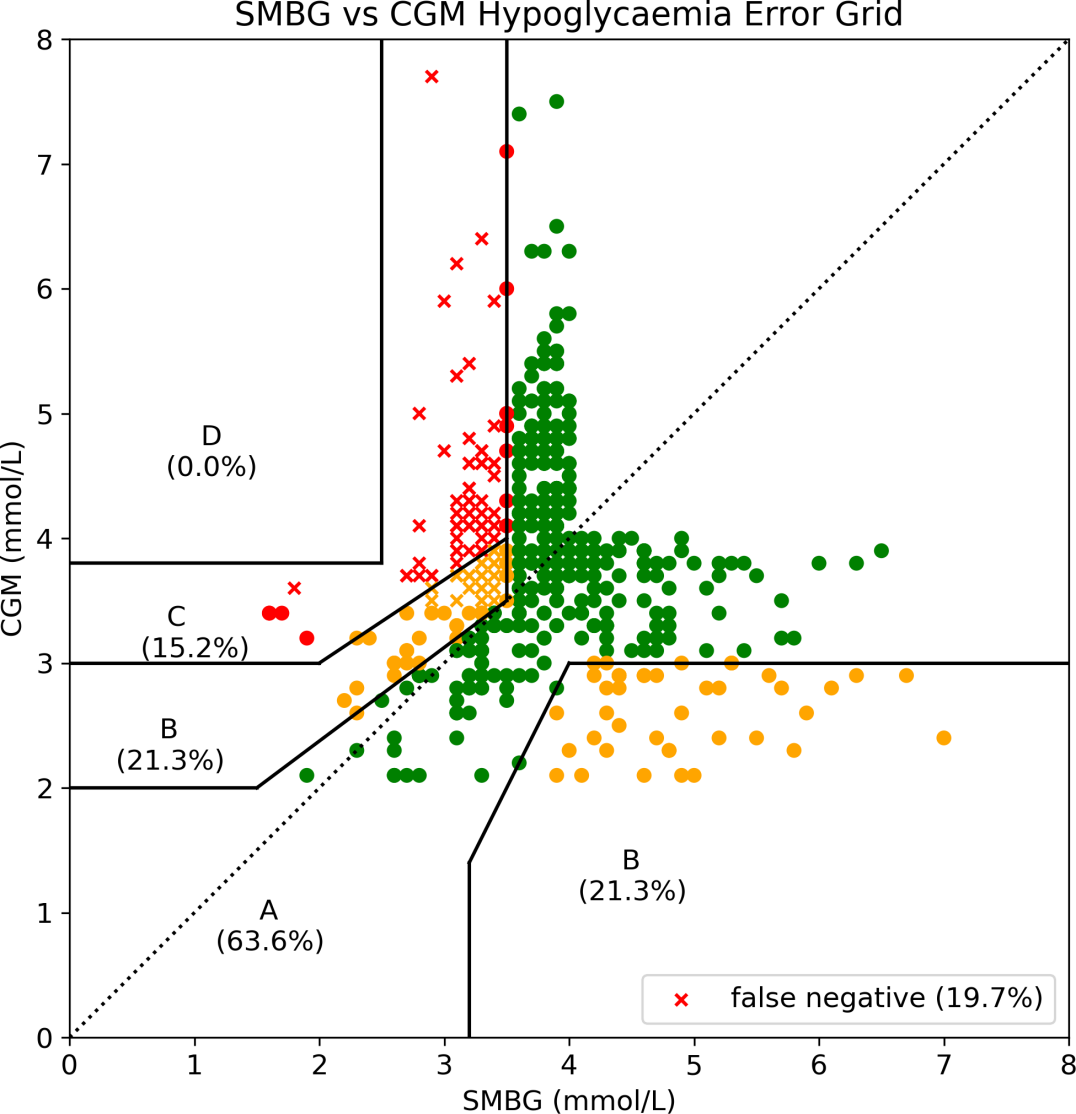
Hypoglycaemia Error Grid plotted with paired values restricted to those in which at least one value was <4mmol/L. It is possible to immediately appreciate the increase in values categorised as slight to moderate risk when the dataset is restricted to that of interest to a patient living with CHI.

We therefore restricted the dataset to those pairs represented by combinations 1-3. This resulted in 528 paired values and these are plotted on the HEG in **Figure 7** and detailed further here and in **Table 3**. Using this new dataset, mean difference was +0.08mmol/L, MAD was 0.89mmol/L and MARD was 23.2% demonstrating a further reduction in accuracy within the area of greatest interest and practicality.

We present the first use of our novel, consensus based HEG designed specifically to analyse the risk of CGM inaccuracies for patients with CHI (**Figure 6D**). This error grid presents risk levels specific to the use of CGM in CHI and thus there are no values classed as no risk for missed hypoglycaemia. This error grid has the added functionality of plotting false negative values as crosses instead of dots, thereby clearly defining this risk category important to the management of patients with hypoglycaemia. False negatives never fall in the no risk area (A) with no values in the highest risk (D) area.

The relative frequencies of the risks reported by the three error grids are presented in **Table 3**.

## Discussion

The utility of CGM in childhood hypoglycaemia disorders has not been fully established. While the application in diabetes is clear (20–22), the use of CGM in hypoglycaemia disorders like CHI is currently being explored. There has been concern over the accuracy of CGM sensor performance in the hypoglycaemia range, with observational studies reporting on false positive errors (12) by graphical methods that categorised errors into discrete grids (13). However, existing error grids do not accurately represent the risk of CGM inaccuracy for patients with CHI as they are designed to test accuracy in patients with diabetes. They underemphasise the risk of missed hypoglycaemia and overemphasise the risk of missed hyperglycaemia as well as attributing importance to higher values of glucose that are of no relevance to the majority of those with CHI. Hence, there is a clear need to develop a hypoglycaemia specific error grid that identifies the risk of missing hypoglycaemia in a clinically meaningful way. Our HEG provides an appropriate tool to understand the true risk of misidentifying hypoglycaemia by rapidly evolving technologies incorporating CGM.

The HEG provides risk identification that is specific to hypoglycaemia in contrast to other available error grids designed for use in diabetes. The consensus for the development of the HEG has been limited to UK specialists given an established network (the CHI Special Interest Group) within the National Health Service in the UK. Our study is therefore limited by the lack of an international consensus. However, at present, there is no uniform international consensus guiding the practical management of hypoglycaemia scenarios in CHI. Further, our proposed grid is not limited for use in the UK and is freely testable for other consensus opinions. To facilitate alternative iterations and interpretations, the code is now freely available on Github (19) with descriptions on: how to plot paired values within the grid; adapt this for alternative thresholds of hypoglycaemia; and calculate the percentage of values lying within each risk category. The study team are available to facilitate this analysis for any research teams who wish to contribute.

To demonstrate the utility of our new HEG we have also presented a large dataset of paired CGM vs SMBG values in a population of CHI patients in free-living conditions. This is also the first evaluation in patients with CHI of the accuracy of the Dexcom G6, a device which is increasingly used in this patient group but has not been previously assessed for accuracy. The average over-reading of the G6 device for all values of glucose is of concern given the level of inaccuracy of almost ± 1 mmol/L for every reading and the worsening of accuracy below glucose levels of 5mmol/L. Although, CGM hypoglycemia detection rates were marginally higher over a 30 minute window around an SMBG hypoglycaemia, our findings suggest that Dexcom G6 CGM (vis a vis SMBG) is inapplicable as a standalone tool for the detection of hypoglycaemia.

A strength of our study is the ability to pair SMBG and CGM values using a consistent and time aligned approach. We paired SMBG to the nearest 5 minute CGM value either before or after the hypoglycaemia event. An alternative strategy proposed by CGM device manufacturers is to find the closest CGM value *after* each SMBG value. This latter technique only includes CGM values with timestamps after the matched SMBG value and thus is prone to report better accuracy due to the time lag on the CGM device. However, most authors describing paediatric use of CGM had opted for pairing of the closest CGM value; thus our method is consistent with previous use of CGM pairing strategies for hypoglycaemia. This method also increases clinical relevance: it reduces the mean time difference between values; excluding values before the SMBG is counter intuitive as the value the patient will be looking at is likely to be the one before (and to have triggered) the SMBG value.

Given that hypoglycaemia sensitivity is low (44%) and false negatives are high (104 values (7.2%)) it is vital that clinicians consider error margins when using CGM in clinical practice. When paired values are restricted to the area of interest for those with CHI (<4mmol/L), the percentage of values classed as moderate risk is 15%, further suggesting that sole reliance on current CGM devices to detect hypoglycaemia for patients with CHI is potentially unsafe. The use of all error grids are somewhat limited in the analysis of CGM accuracy as they do not account for the trend information provided to users and this must be taken into account when interpreting the above results.

Somewhat reassuringly, the frequent advice given to patients that they can trust CGM accuracy when in the normal range was found to be largely true for values >5mmol/L where patients were very unlikely to be hypoglycaemic. For CGM values >4mmol/L and >5mmol/L the incidence of hypoglycaemia was 4% and 1% respectively, although these values may be artificially low due to minimal incentive to check an SMBG when CGM is reading normal and thus small numbers. **Table 2** can be used to facilitate discussions with patients and families to explain the risk of missed hypoglycaemia. It is also important for those families requesting CGM to understand the average divergence from SMBG is almost 1mmol/L and the resulting clinical implications if solely relying on CGM to identify hypoglycaemia. Nonetheless, low accuracy does not detract from utilising CGM in alternative ways, for instance in the analysis of trends and digital phenotypes as has been reported in both diabetes (23, 24) and CHI (17, 25). Finally, it is of the utmost importance that families’ opinions on their experiences of CGM devices are sought and documented, as has been achieved for the first time recently (26).

The HEG has the potential to engineer the design and development of next generation CGM sensors that are more specific to hypoglycaemia, assuming that manufacturers design algorithms to target hypoglycaemia rather than hyperglycaemia (27). While the majority of patients using CGM will have diabetes, the number of patients using CGM as part of a non-diabetes condition is increasing; therefore, the HEG is a prompt for device manufacturers to include specific application in hypoglycaemia in their sensor and algorithm development strategy.

## Conclusion

The Hypoglycaemia Error Grid (HEG) developed by a consensus of paediatric endocrinologists in the UK is a specific hypoglycaemia tool to identify the clinical risk in hypoglycaemia disorders such as CHI. The tool is specific for application in hypoglycaemia and is superior to available error grids designed for use in diabetes. Using the HEG, analysis of the largest CHI specific paired CGM dataset demonstrated insufficient accuracy and low rates of hypoglycaemia detection. Thus, at present the routine use of CGM as a standalone diagnostic tool in patients with CHI is not recommended. The application of CGM in hypoglycaemia disorders requires further research and development.

## Data availability statement

The raw data supporting the conclusions of this article will be made available by the authors, without undue reservation.

## Ethics statement

The studies involving human participants were reviewed and approved by REC and HRA ethical approval (REC reference 07/H1010/88). Written informed consent to participate in this study was provided by the participants’ legal guardian/next of kin.

## Author contributions

CW designed and ran the data collection process as well as designing the questionnaire for the error grid, collating information, writing the code for generation of the grid and developing the grid. CW also drafted the first version of the manuscript. MD, MS-E, SH, PN, AD, SS, and IB contributed to the development of the error grid and reviewed and approved the final version of the manuscript.

## Funding

This work was partially supported by Health Innovation Manchester Momentum Fund.

## Acknowledgments

The authors would like to thank the paediatric endocrinology consultants of Royal Manchester Children’s Hospital, Alder Hey Children’s Hospital and Great Ormond Street Hospital who kindly contributed to the generation of the Hypoglycaemia Error Grid by completing a questionnaire.

## Conflict of interest

The authors declare that the research was conducted in the absence of any commercial or financial relationships that could be construed as a potential conflict of interest.

## Publisher’s note

All claims expressed in this article are solely those of the authors and do not necessarily represent those of their affiliated organizations, or those of the publisher, the editors and the reviewers. Any product that may be evaluated in this article, or claim that may be made by its manufacturer, is not guaranteed or endorsed by the publisher.
