# Supplementary material for: The hypoglycaemia error grid: A UK-wide consensus on CGM accuracy assessment in hyperinsulinism
Source: Front Endocrinol (Lausanne). 2022 Nov 2;13:1016072. doi: 10.3389/fendo.2022.1016072 (PMC9666389; doi:10.3389/fendo.2022.1016072)
Supplement: Supplementary file 1 [file DataSheet_1.docx]

# Appendix 1

## A copy of the questionnaire sent to consultants

Please provide what you think should be the glucose ranges for patients (irrespective of symptoms) with HI for each of the codes and actions below. CGM = continuous glucose monitoring. SMBG = Self monitored blood glucose (fingerprick)

| BG ranges and associated actions | | |
| --- | --- | --- |
| Code | Action | Suggested glucose range (e.g. 0.0-2.0) |
| 1 | Emergency treatment for hypo (e.g. IM glucagon) |  |
| 2 | Quick treatment for hypo (e.g. glucogel) |  |
| 3 | Eat if due meal and/or check again in 10 mins |  |
| 4 | No action required but close monitoring |  |
| 5 | No action required. No repeat monitoring. |  |

We will now ask you to assign a level of risk to 25 categories in the table below based on the codes you have just assigned. The degrees of risk are described before the table. Please note that **the risk is of the discrepancy between CGM and SMBG, not of the glucose value itself**. Thus there is no risk when the methods report the same value and the codes are the same. As such, the table is prepopulated with risk A (none) in these spaces. Risk is likely to be highest (D) for e.g. at times when CGM reports a high value (e.g. Code 5) but in fact SMBG value is low (code 1) and thus patient is unaware of a severe hypo occurring.

This assumes the patient is acting simply on values presented on CGM meter (rather than symptoms) and the true value is that from the SMBG.

Degrees of risk:

A: None, B: slight, C: moderate, D: severe/dangerous

| Degree of risk | | | |
| --- | --- | --- | --- |
| CGM is reading  Code | Action patient is therefore taking | Actual (SMBG value) is  Code | Degree of risk  (A, B, C or D) |
| 1 | Emergency treatment for hypo (e.g. IM glucagon) | 1 | A |
|  |  | 2 |  |
|  |  | 3 |  |
|  |  | 4 |  |
|  |  | 5 |  |
| 2 | Quick treatment for hypo (e.g. glucogel) | 1 |  |
|  |  | 2 | A |
|  |  | 3 |  |
|  |  | 4 |  |
|  |  | 5 |  |
| 3 | Eat if due meal and/or check again in 10 mins | 1 |  |
|  |  | 2 |  |
|  |  | 3 | A |
|  |  | 4 |  |
|  |  | 5 |  |
| 4 | No action required but close monitoring | 1 |  |
|  |  | 2 |  |
|  |  | 3 |  |
|  |  | 4 | A |
|  |  | 5 |  |
| 5 | No action required. No repeat monitoring | 1 |  |
|  |  | 2 |  |
|  |  | 3 |  |
|  |  | 4 |  |
|  |  | 5 | A |
